# Predictive factors of recurrence in spontaneous bacterial peritonitis in Tunisian patients with cirrhosis

**DOI:** 10.2144/fsoa-2023-0016

**Published:** 2023-04-17

**Authors:** Manel Moalla, Nour Elleuch, Wafa Dahmani, Aya Hammami, Wafa Ben Ameur, Aida Ben Slama, Ahlem Brahem, Salem Ajmi, Mehdi Ksiaa, Hanen Jaziri, Ali Jmaa

**Affiliations:** 1 Department of gastroenterology, Sahloul University Hospital, Sousse, 4054, Tunisia

**Keywords:** cirrhosis, prognosis, recurrence, spontaneous bacterial peritonitis, Tunisia

## Abstract

**Introduction:**

Recurrent spontaneous bacterial peritonitis (SBP) in patients with cirrhosis is associated with poor prognosis.

**Aim:**

To assess the prevalence and the risk factors for recurrence and to evaluate its impact on the prognosis.

**Materials & methods:**

We conducted a retrospective study including patients with cirrhosis having a first episode of SBP.

**Results:**

A recurrence of SBP was identified in 43.4% of the patients who survived after a first episode of SBP. The mean time to onset of the first SBP recurrence from the first episode was 32 days. Recurrence factors were endoscopic hypertensive signs, a positive ascites culture, diarrhea and the MELD score.

**Conclusion:**

There was no impact on survival of recurrent SBP compared with the first SBP episode.

## Plain language summary

Recurrent spontaneous bacterial peritonitis (SBP) in patients with cirrhosis is associated with poor prognosis. The aims of this study were to assess the prevalence and the risk factors for recurrence and to evaluate its impact on the prognosis. Thus, we conducted a retrospective study including patients with cirrhosis having a first episode of SBP. A recurrence of SBP was identified in 43.4% of the patients who survived after a first episode of SBP. Recurrence factors were endoscopic hypertensive signs, a positive ascites culture, diarrhea and the MELD score.

## Introduction & objectives

Spontaneous bacterial peritonitis (SBP) is a specific complication of cirrhosis [1]. Its prevalence in patients hospitalized for ascites is estimated between 10 and 30%. It constitutes a diagnostic and therapeutic life-threatening emergency with a hospital mortality approaching 20–40% and 1 year mortality estimated at 60% [1,2]. In patients who have survived a first SBP, the cumulative risk of recurrence at 1 year can reach 70% in the absence of secondary prophylaxis and 20% under prophylactic treatment [3].

Few studies have investigated the predictive factors of recurrence and survival after a first episode of SBP. In fact, identifying these risk factors could improve the management of these patients by selecting those who should be strictly monitored and likely candidates for digestive tract selective decontamination which consists in a short course (4 days) of parenteral antibiotics to control primary endogenous microbes then enteral antimicrobials, in other words, polymyxin E, tobramycin and amphotericin B to control secondary oropharyngeal and intestinal carriage, and subsequent secondary endogenous infections plus high levels of hygiene and surveillance of throat and rectum cultures on admission and, afterward, twice weekly to monitor the effectiveness of digestive tract selective decontamination and to detect resistance at an early stage [4].

The aims of our study were to:

- Determine the predictive factors of recurrence after a first episode of SBP.
- Investigate the overall survival in these patients.

## Materials & methods

We conducted a retrospective study in the gastroenterology department from January 2005 to December 2018, collecting all the files of patients with cirrhosis who were hospitalized for a first episode of SBP. The diagnosis of cirrhosis was established according to biochemical findings, abdominal ultrasonography, histopathological evaluation of liver biopsy as well as endoscopic results suggesting portal hypertension with other variables reporting liver cirrhosis.

The SBP was defined by a neutrophil count greater than 250 cells per mm^3^ in the ascites fluid [1].

### Inclusion criteria

Patients with cirrhosis having a first episode of SBP were included in our study.

### Non inclusion criteria

We did not include patients with bacterascites defined by a positive culture of ascites fluid associated with a neutrophil count <250 cells per mm^3^ in the ascites fluid, patients presenting an empyema or bacterial peritonitis secondary to an intra-abdominal organ perforation and patients having an hepatocellular carcinoma (HCC) or another malignant tumor at the moment of diagnosis of first SBP.

### Exclusion criteria

Patients with a follow-up of less than 6 months were excluded as well as patients who developed HCC or another malignant tumor during the follow-up period.

The management of the SBP was carried out according to international guidelines [5].

First-line antibiotic therapy was based on third-generation cephalosporins, mainly cefotaxime, fluoroquinolones (ofloxacin) or amoxcillin clavulanic acid and albumin infusion at a dose of 1.5 g/kg at day 1 and 1 g/kg at day 3 according to EASL guidelines [5,6].

A second paracentesis was done 48 h after starting antibiotics to check the response to therapy.

Prophylactic therapy with ciprofloxacin 750 mg one tablet per week was prescribed for all patients who survived the first SBP episode. We did not prescribe rifaximin for prophylactic therapy despite some promising evidence, since its use is not yet be recommended as an alternative to norfloxacin for secondary prophylaxis of SBP according to EASL guidelines [5].

## Data collection

For each patient, the following data were collected: demographic, clinical data and laboratory counts of white blood cells, neutrophils, lymphocytes, platelets.

## Statistical analysis

We compared the two groups of patients: with and without recurrence of SBP in order to identify the predictive factors of its recurrence.

Statistical analysis was performed by the 20th version of SPSS for Windows.

Descriptive analysis was performed.

Determination of predictive factors of recurrence was first based on univariate analysis using Student’s test for quantitative variables, Pearson Chi-square test and Fisher exact test for qualitative variables and by calculating the odds ratio (OR) as a measure of risk, as well as their confidence intervals (CI) after transforming the quantitative variables into qualitative variables according to the cut-off values identified by the ROC curves.

The univariate analysis was supplemented by multivariate logistic regression in order to identify independent predictive factors of SBP recurrence.

Survival after a first episode of SBP, as well as survival after recurrence and cause of death were specified using the Kaplan–Meier method.

A p-value of less than 0.05 was considered to be statistically significant.

## Results

### Patient characteristics

Our study involved 63 patients with cirrhosis having a first SBP episode. The average age at the time of diagnosis of SBP was 62 years [29–92 years]. The sex ratio (men/women) was 2. Twenty patients (31.7%) had at least one comorbidity. The causes of cirrhosis were HBV infection (37 patients: 58.7%), HCV infection (eight patients: 12.6%), non alcoholic steatohepatitis (five patients: 7.9%), alcohol consumption (four patients: 7.5%) and autoimmune diseases (three patients: 4.7%). Cirrhosis was classified as Child B and C in 49 (77.7%) and 14 patients (22.2%), respectively. The median MELD score was 16 (8–34). Forty-five patients (71.4%) had a MELD score >15.

Forty-five (71.4%) patients had developed ascites at least once before the diagnosis of SBP and 22 (34.9%) had at least one episode of gastrointestinal bleeding. Refractory ascites was already present in 15 patients (23.8%). Two patients had a previous episode of hepatic encephalopathy (3.2%). Three patients had portal thrombosis (4.8%). Esophageal varices were found in all patients. They were grade I, II and III in 5 (7.9%), 42 (66.7%) and 16 (25.4%) patients, respectively. Gastric varices were present in 24 patients (61.9%). Twenty-four patients (38.1%) had hypertensive gastropathy.

Fifteen patients (23.8%) had primary prophylaxis. In all cases, it was ciprofloxacin at a dose of 750 mg per week. The average duration of prophylaxis before diagnosis of the first SBP episode was 8.4 months (2–12 months).

Contributing factors were noted in 27 patients (42.8%) during the 15 days before the occurrence of SBP. Two patients had two contributing factors. These various factors were large volume paracentesis, gastrointestinal bleeding, endoscopy and proton pump inhibitors in, respectively ten, fifteen, two and three patients.

#### Characteristics of the first SBP episode

The diagnosis of SBP was concomitant with the diagnosis of cirrhosis in eight patients (12.7%). SBP occurred within an average of 20.6 months from the diagnosis of cirrhosis [0–108].

Forty-five patients (71.4%) had community acquired SBP, 11 (17.5%) had healthcare associated SBP and 7 (11.1%) had nosocomial SBP. The clinical symptomatology was dominated by fever present in 48 patients (76.2%).

The SBP was associated with the following severity criteria: septic shock in six patients and hepatic encephalopathy in 14 of them. The SBP was completely asymptomatic in six patients.

The different symptoms are summarized in the Table 1.

**Table 1.** Spontaneous bacterial peritonitis symptoms in our patients.

Symptoms	Patients (n)	Percentage (%)
Fever	48	76.2
Abdominal pain	44	69.8
Jaundice	17	27
Encephalopathy	14	22.2
Diarrhea	11	17.5
Sepsis signs	6	9.5
Vomiting	**3**	6.1

Fifteen patients had an associated infection. It was urinary tract infection in eight patients (12.7%), bronchopulmonary infection in five patients (7.9%) and skin infection in two patients (3.2%) notably erysipelas in one patient.

High levels of white blood cell were noted in 41 patients (65.1%) while leukopenia was observed only in two patients (3.2%). Thrombocytopenia defined by a platelets count below 150,000/ml was noted in 43 patients (68.3%). Renal failure was observed in 20 patients (31.2%).

The ascites fluid was cloudy in nine patients (14.3% of cases) and hemorrhagic in six patients (9.5%). The protein content in the ascites fluid varied between 1 g/l and 18 g/l with an average of about 8.7 g/l. Sixty-one patients (96.8%) had a protein level in ascites fluid <15 g/l and 37 patients (58.7%) had a protein level in ascites fluid <10 g/l. Direct bacteriological examination of ascites fluid was positive in nine patients with isolation of *Escherichia coli* (*E. coli*) and *Klebsiella pneumoniae* (*K. pneumoniae*) in four and five patients. respectively. The ascites fluid culture was positive in 14 patients (22.2%): *E. coli* in six patients and *K. pneumoniae* in seven patients.

#### Therapeutic modalities of the first SBP episode

Fifty-seven patients (90.5%) received cefotaxime antibiotics prescribed at a dose of 3–4 g/day for a period ranging from 5 to 10 days.

Five patients (7.9%) received ofloxacin administered at a dose of 400 mg/day for 7–10 days. One patient received amoxicillin clavulanic acid at a dose of 3 g/day for 7–10 days. In all cases, the treatment was administered intravenously exclusively. The average duration of antibiotic therapy was 5 days (2–10 days).

A 20% human albumin infusion was associated with antibiotic therapy in all patients with an average of three vials per day (2–4) for an average of 3 days (2–10).

A favorable response was observed in 56 patients (88.9%). Seven cases of nonresponse to first-line treatment were observed. Second-line antibiotic therapy was mainly based on imipenem, tazocillin and amoxicillin clavulanic acid in three, two and two patients, respectively. This escalation was guided by an antibiogram in two patients.

Thirty-three patients (55%) experienced complications. It was mainly an encephalopathy, hepatorenal syndrome (HRS) in 26 cases (29%), acute renal failure, septic shock and GI bleeding in seventeen, ten and four patients, respectively.

In-hospital mortality was 15.9% (n = 10) and occurred within an average of 7 days (2–10). The causes of death were septic shock, hepatorenal syndrome, hepatic encephalopathy and GI bleeding in three, four, one and one patient, respectively.

Prophylactic therapy with ciprofloxacin 750 mg one tablet per week was prescribed for all patients who survived the first SBP episode (n = 53). This treatment was taken regularly by 42 patients (79.2%).

#### First recurrence of SBP

SBP recurrence was noted in 23 of the 54 patients who survived after a first SBP episode (42.6%). The mean time to onset of the first SBP recurrence was 32 days (4–126). Antibiotic therapy of the first recurrence of SBP was based mainly on third generation cephalosporins including cefotaxime, ofloxacin and imipenem in fourteen, two and seven patients, respectively. The course was favorable in 21 patients (91.3%). The number of SBP recurrences varied between 0 and 3.

#### Predictive factors of recurrence of SBP

##### Predictive factors of recurrence of SBP in univariate analysis were

- The presence of hypertensive gastropathy (HTG) (p = 0.005, OR = 6.8)
- The presence of severe esophageal varices (p = 0.046, OR = 2.58)
- The presence of gastric varices (p = 0.005, OR = 5.08)
- A positive ascites culture (p = 0.001, OR = 3.8)
- Diarrhea (p = 0.001, OR = 2.7)
- Secondary prophylactic antibiotic intake (p = 0.005, OR = 3.85)
- Bilirubin with a cut off of 54.5 μmol/l with a sensitivity and specificity of 70 and 76.8%, respectively, and an area under the curve of 0.73 (p = 0.01)
- MELD score (p = 0.023) with a cut off of 17 with a sensitivity and specificity of 91 and 66.7%, respectively, and an area under the curve of 0.728 (p = 0.014)

Low albumin-level, low platelet count and high hemoglobin were significantly associated with recurrent SBP with p of 0.009, 0.001 and 0.013, respectively. A lower age at diagnosis of SBP and a longer interval between diagnosis of cirrhosis and SBP were significantly associated with recurrent SBP with p-values of 0.033 and 0.008, respectively.

The results of univariate study were summarized in (Tables 2 & 3).

**Table 2.** Predictive factors of recurrence of spontaneous bacterial peritonitis in univariate analysis (qualitative variables).

Recurrent SBP				
Parameters	Yes (n = 23)	No (n = 30)	p-value	OR (CI = 95%)
Gender: Men/women	15/8	19/11	0.88	
Comorbidity	5	6	0.87	
Diabetes	5	3	0.23	
Hypertension	0	6	0.02	
Proton pump inhibitors	8	17	0.176	
Beta blockers	17	27	0.122	
Viral cirrhosis	17	20	0,56	
Child B	5	8	0.944	
Child C	18	22	0.67	
Hepatic encephalopathy	0	1	0.377	
Gastrointestinal bleeding	6	13	0.194	
Hypertensive gastropathy	19	4	**0.005**	**6.8**
Severe esophageal varices	23	27	**0.046**	**2.6**
Gastric varices	18	5	**0.005**	**5.1**
Healthcare associated SBP	2	8	0.644	
Community acquired SBP	19	20	0.545	
Nosocomial SBP	2	2	0.483	
Positive ascites culture	11	2	**0.001**	**3.8**
Fever	17	23	0.81	
Diarrhea	8	2	**0.01**	**2.7**
Failure of the first line antibiotic	0	2	0.207	
Secondary prophylactic antibiotic	14	28	**0.005**	**3.9**

The figures in bold represent significant results: p < 0.05.

OR: Odds ratio; SBP: Spontaneous bacterial peritonitis.

**Table 3.** Predictive factors of recurrence of spontaneous bacterial peritonitis in univariate analysis (quantitative variables).

Recurrent SBP			
Parameters	Yes (n = 23)	No (n = 30)	p-value
Age at the diagnosis of the first episode of SBP (years)	56.4 ± 9.9	63.7 ± 13.5	**0,033**
Delay between the diagnosis of cirrhosis and SBP (months)	34.3 ± 39.2	11.8 ± 18.5	**0.008**
Leucocytes (cell/mm^3^)	10412 ± 3547	11440 ± 4512	0.167
Hemoglobin (g/dl)	10.7 ± 1.3	8.9 ± 3.4	**0.013**
Platelets (cell/mm^3^)	81 217 ± 41 182	140 266 ± 76 072	**0.001**
CRP (mg/l)	83.3 ± 33.2	93.8 ± 45.9	0.358
Serum creatinin (μmol/l)	91.8 ± 59	95.0 ± 47.9	0.784
Serum urea (mg/l)	6.9 ± 3.8	8.1 ± 6.1	0.414
Natremia (mmol/l)	133.7 ± 5.1	134.5 ± 4.8	0.608
Bilirubin (μmol/l)	88.9 ± 62.9	47.5 ± 49.8	**0.010**
Serum Albumin (g/l)	20.9 ± 4.3	25.3 ± 6.7	**0.009**
Prothrombin time (%)	46.7 ± 12.5	50.9 ± 16.3	0.318
MELD score	21.9 ± 5.4	17.5 ± 6.8	**0.014**
Ascitic protein level (g/l)	7.9 ± 2.4	9.8 ± 3.9	**0.053**
Ascitic leucocytes level (cell/mm^3^)	3450 ± 4408	1799 ± 1399	**0.056**
Ascitic neutrophils (cell/mm^3^)	2824.9 ± 3643	1443 ± 1287	**0.059**

The figures in bold represent significant results: p < 0.05.

CRP: C-reactive protein; MELD: Model for end stage liver disease; SBP: Spontaneous bacterial peritonitis.

##### In multivariate analysis, the independent factors associated with a SBP recurrence were

- The presence of gastric varices (p = 0.049)
- The presence of HTG (p = 0.011)
- A positive culture (p = 0.017)
- The age of diagnosis of SBP (p = 0.013)
- The presence of diarrhea at admission (p = 0.057)

#### Survival of patients

The recurrence of SBP was associated with a decrease in survival, going from 15.2 months in the absence of recurrence to 13.8 months with no significant difference between the two groups (p = 0.8).

**Figure 1.**
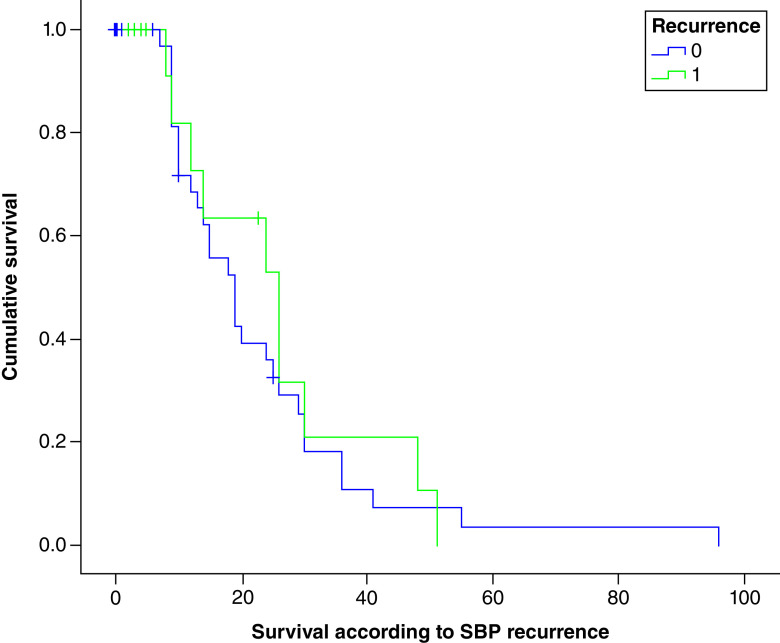
Survival curve according to spontaneous bacterial peritonitis recurrence. SBP: Spontaneous bacterial peritonitis.

The SBP recurrence survival curve is shown in Figure 1.

## Discussion

In our study, a recurrence of SBP was identified in 43.4% of the patients who survived after a first episode of SBP and this despite taking a secondary antibiotic prophylaxis noted in 69.6% of the cases. The mean time to onset of the first SBP recurrence from the first episode was 32 days (range: 4–126). The independent factors significantly associated with the occurrence of SBP recurrence were HTG, the presence of gastric varices, a positive culture, the presence of diarrhea at admission and a low age of diagnosis of SBP were independent factors for SBP recurrence.

The recurrence of SBP was associated with a decrease in survival, going from 15.2 months in the absence of recurrence to 13.8 months with no significant difference between the two groups (p = 0.8).

### Prevalence of recurrent SBP in cirrhotic patients

SBP recurrence has been little studied. Historically, it was described by Tito *et al.* in 1988 in 43% of survivors at 6 months, 69% at 1 year and 74% at 2 years in the absence of prophylaxis [7]. This rate is reduced to around 20% with norfloxacin in the randomized trial carried out by Gines *et al.* in 1990 [3,8]. This trial excluded patients with very severe liver disease with prothrombin time (PT) <25%, bilirubin level >10 mg/l or creatinine level >2 mg/dl or with a history of chronic encephalopathy or with HCC and included 75 patients having survived a first SBP episode [3]. Since this trial, very few studies have investigated the prevalence of recurrent SBP in patients with cirrhosis on norfloxacin antibiotic prophylaxis. Huang *et al.* conducted a retrospective study including 146 cirrhotic patients with a first SBP episode [9]. Among these patients, 89 survived and 38 had a recurrence (42.7%). Similar results were obtained in the unicentric prospective study of Nair *et al.* published in 2018 where a recurrence of SBP was noted in 40.5% of patients [10]. A lower prevalence of recurrent SBP was observed in the study of Marciano *et al.* published in 2019, which included 115 patients with cirrhosis who survived a first SBP episode and who were on norfloxacin antibiotic prophylaxis [11]. In our study, SBP recurrence was noted in 23 patients out of 53 who survived after a first episode of SBP (43.4%).

### Risk factors of recurrence after a first SBP episode

Despite the few studies confirming these data, experts recommend instituting a long term secondary prophylaxis with norfloxacin 400 mg/day after a first SBP episode. If there is no indication for transplantation, antibiotic prophylaxis is recommended for life, or until complete disappearance of ascites [5]. As with any prolonged use of antibiotics, the emergence of quinolone resistant germs is to be feared. Thus, better knowledge of the predictors of recurrence could identify a high-risk group needing antibiotic prophylaxis avoiding overuse of antibiotics. So far, few studies have investigated the risk factors of recurrence and our literature research has isolated only five studies about this subject.

In the study by Tito *et al.*, the independent predictive factors of SBP recurrence were the low protein levels in ascites and low PT. This has led the authors to suggest that patients with advanced liver disease reflected by high bilirubin, low PT and low ascitic total protein concentration are more likely to recur [7]. The elements of this analysis were studied in our series and were identified as predictive factors of recurrent SBP in the univariate study and not found in the multivariate study. In the study by Jamil *et al.*, age >55 years, bilirubin >1 mg/l and a history of urinary tract infection were independent factors of SBP recurrence. Conversely, HBV related cirrhosis was a protective factor against recurrence (OR = 0.31) [12].

In the study by Huang *et al.* the predictive factors of recurrence were an albumin level <28.5 g/l with a sensitivity of 70,2%, specificity of 76.3%, as well as taking beta-blockers. In multivariate analysis, only beta-blockers intake was an independent factor of recurrent SBP (p = 0.048) [9].

In the study published in 2018 by Nair *et al.*, the presence of fever on admission, hepatic encephalopathy, diuretics, antibiotic prophylaxis, recent use of antibiotics (<30 days), iterative large-volume paracentesis, Child Pugh score C and presence of HTG were significantly associated with recurrent SBP [10]. In multivariate analysis, HTG was an independent factor of SBP recurrence (p = 0.001) which consolidates the results of our study: Indeed, both the presence of HTG and gastric varices were identified as independent predictive factors of SBP recurrence. These results could be explained by the hypothesis that portal hypertension plays a role in bacterial translocation and therefore the prediction of bacterial infections during decompensated cirrhosis [10].

In Marciano *et al.* study, the independent predictive factor of recurrence identified was male gender (OR = 2.52) [11]. This factor was not identified as a predictive factor of recurrence in our study.

### Prognosis of recurrent SBP

The prognosis has improved considerably since the first description of SBP. In-hospital mortality decreased from 100% in 1960 to 60–70% between the 1970s and 1980 to 30% or less in the last 10 years [13]. This is likely due to the precocity of diagnosis and the initiation of effective therapy [13]. If remission is most often obtained, in-hospital mortality is always around 30%, related to non directly infectious causes: hepatorenal syndrome, GI bleeding, end-stage liver disease [14,15]. While the prognostic impact of a first SBP episode is currently well known, the impact of recurrent SBP on survival is little known and has been little studied.

In the Spanish study by Tito *et al.* the probability of survival for patients with SBP recurrence was 38% at 1 year, 27% at 2 years and 16% at 3 years of follow-up [7]. Similarly, the SBP recurrence was significantly associated with a decrease in survival compared with patients with cirrhosis without recurrence (p < 0.01) [7]. Similar results were obtained in the study of Nair *et al.* [10]. Indeed, this study showed that recurrent SBP was associated with higher mortality (p = 0.009) and lower survival (p = 0.04) compared with the first SBP episode [10].

In Huang *et al.* and Marciano *et al.* studies, recurrent SBP was associated with a lower survival but no significant difference with the non relapsing group [9,11]. These results were consistent with the results of our study. Indeed, SBP recurrence was associated with a decrease in survival, going from 15.2 months in the absence of recurrence to 13.8 months in the case of recurrence with no significant difference between the two groups (p = 0.8).

## Limitations

The affordability of albumin was a limitation in our study since we could not afford 1.5 g/kg for some patients which. The second limitation was the unavailability of norfloxacin in our country which led us to prescribe ciprofloxacine for secondary prophylaxis. The third limitation is that we did not investigate vitamin D supplementation on SBP recurrence prevention despite that some studies have proven its role in preventing bacterial translocation.

## Conclusion

Recent data on the natural history of patients who have survived a first episode of SBP are limited. Recurrent SBP is an emerging problem while its prevalence, risk factors and prognostic impact remain poorly understood. However, knowing these factors could select the patients who must be under strict surveillance and probably candidates for selective intestinal decontamination.

At the end of this study, we were able to estimate the prevalence of recurrent SBP during cirrhosis and its occurrence predictors. In our study, recurrent SBP was relatively frequent since it affected half of the patients who survived the first episode. The presence of portal hypertensive gastropathy and gastric varices were associated with recurrent SBP. Regarding the prognosis, we confirmed that recurrent SBP had a poor prognosis. Impact on survival compared with the first SBP episode has not been proven.

### What is already known on this topic

- Patients with cirrhosis with a recurrent SBP have poor prognosis.
- The absence of secondary prophylaxis is a well know predictive factor of SBP recurrence.

### What this study adds

- The presence of portal hypertensive gastropathy and gastric varices were associated with recurrent SBP.
- There was no significant difference in the survival between patients having a first SBP or a recurrent SBP.

**Summary points**

- Patients with cirrhosis with a recurrent spontaneous bacterial peritonitis (SBP) have poor prognosis.
- The absence of secondary prophylaxis is a well know predictive factor of SBP recurrence.
- The presence of portal hypertensive gastropathy and gastric varices were associated with recurrent SBP.
- There was no significant difference in the survival between patients having a first SBP or a recurrent SBP.
